# Involvement of lysosomal dysfunction in silver nanoparticle-induced cellular damage in A549 human lung alveolar epithelial cells

**DOI:** 10.1186/s12995-016-0090-0

**Published:** 2016-01-12

**Authors:** Takamitsu Miyayama, Masato Matsuoka

**Affiliations:** Department of Hygiene and Public Health I, Tokyo Women’s Medical University, 8-1 Kawada-cho, Shinjuku-ku, Tokyo 162-8666 Japan

**Keywords:** Silver nanoparticles, A549 cells, Cellular damage, Bafilomycin A1, Lysosomes, Metallothionein

## Abstract

**Background:**

While silver nanoparticles (AgNPs) are widely used in consumer and medical products, the mechanism by which AgNPs cause pulmonary cytotoxicity is not clear. AgNP agglomerates are found in endo-lysosomal structures within the cytoplasm of treated cells. In this study, the functional role of lysosomes in AgNP-induced cellular damage was examined in A549 human lung alveolar epithelial cells. We evaluated the intracellular distribution of AgNPs, lysosomal pH, cellular viability, Ag dissolution, and metallothionein (MT) mRNA levels in AgNP-exposed A549 cells that were treated with bafilomycin A1, the lysosomal acidification inhibitor.

**Findings:**

Exposure of A549 cells to citrate-coated AgNPs (20 nm diameter) for 24 h induced cellular damage and cell death at 100 and 200 μg Ag/ml, respectively. Confocal laser microscopic examination of LysoTracker-stained cells showed that AgNPs colocalized with lysosomes and their agglomeration increased in a dose-dependent manner (50–200 μg Ag/ml). In addition, the fluorescence signals of LysoTracker were reduced following exposure to AgNPs, suggesting the elevation of lysosomal pH. Treatment of A549 cells with 200 nM bafilomycin A1 and AgNPs (50 μg Ag/ml) further reduced the fluorescence signals of LysoTracker. AgNP-induced cell death was also increased by bafilomycin A1 treatment. Finally, treatment with bafilomycin A1 suppressed the dissolution of Ag and decreased the mRNA expression levels of MT-I and MT-II following exposure to AgNPs.

**Conclusions:**

The perturbation of lysosomal pH by AgNP exposure may play a role in AgNP agglomeration and subsequent cellular damage in A549 cells.

## Findings

### Background

Silver nanoparticles (AgNPs) are widely used in consumer and medical products; however, much attention is now being paid to their adverse health effects in humans. Inhalation is considered the most important route of exposure for nanoparticles, including AgNPs [1]. Because repetitive inhalation of AgNPs induces pulmonary damage in animal models [2, 3], it is important that we clarify the mechanism underlying AgNP pulmonary cytotoxicity.

Morphological investigations using confocal laser microscopy and transmission electron microscopy (TEM) have shown that AgNPs are taken up by a variety of cell types and deposited as agglomerates/aggregates in endosomes or lysosomes within the cytoplasm [4–8]. However, to the best of our knowledge, the toxicological significance of lysosomal dysfunction in AgNP-exposed lung epithelial cells has not been examined.

Lysosomes are membrane-bound organelles containing hydrolases that function in the degradation of macromolecules delivered via the endocytic, phagocytic, and autophagic pathways [9]. The lumenal environment is maintained at a pH of 4.6–5.0 by proton-pumping ATPase (H^+^-ATPase) [10]. Bafilomycin A1, a macrolide antibiotic isolated from *Streptomyces* sp., is a highly specific inhibitor of vacuolar H^+^-ATPase [11]. This substance has been reported to increase lysosomal pH and induce lysosomal dysfunction in cultured cells [12]. In the present study, we examined the effects of bafilomycin A1 treatment on cellular damage induced by citrate-coated AgNP (20 nm diameter) exposure in A549 human lung alveolar epithelial cells. We evaluated the intracellular distribution of AgNPs, lysosomal pH, cellular viability, Ag dissolution, and expression of the Ag^+^-inducible metallothionein (MT) gene in AgNP-exposed A549 cells in the presence of bafilomycin A1.

## Methods

### Cell culture and treatments

**Fig. 1:**
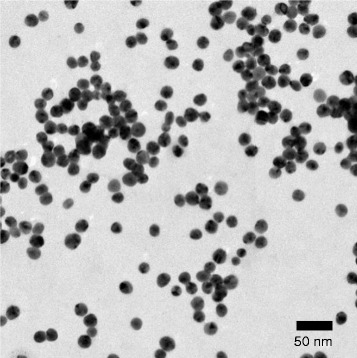
Transmission electron microscopy image of 20 nm AgNPs. AgNPs suspension in methanol were placed onto a collodion membrane-attached sheet mesh (150-A) and dried at room temperature. Scale bar, 50 nm

A549 cells (Japan Health Sciences Foundation, Osaka, Japan) were grown in minimum essential medium with non-essential amino acids supplemented with 10 % heat-inactivated fetal bovine serum, 100 U/ml penicillin, and 100 μg/ml streptomycin (GIBCO, Invitrogen Corp., Carlsbad, CA, USA) in a humidified atmosphere of 5 % CO_2_ and 95 % air at 37 °C. Exponentially growing A549 cells were seeded on 6-well culture plates, 96-well culture plates, or Imaging Chamber CG slides and cultured for 24 h before each experiment. Citrate-capped 20 nm AgNPs (Citrate NanoXact™ Silver) were obtained from nanoComposix (San Diego, CA, USA). A TEM image of the AgNPs used is shown in Fig. 1. The particle diameter and the zeta-potential of these AgNPs in culture medium containing 1 % albumin were 36.6 ± 21.4 nm (mean ± standard deviation, *n* = 70) and −15.4 mV, respectively. Cells were exposed to the appropriate concentrations of well-dispersed AgNPs (50–1000 μg Ag/ml) for 24 h in 1 % albumin-containing culture medium. Bafilomycin A1 (LC Laboratories, Woburn, MA, USA) was dissolved in dimethyl sulfoxide (DMSO). Cells were incubated in culture medium containing 0.4 % DMSO or 200 nM bafilomycin A1 for 24 h before treating with AgNPs for an additional 24 h.

### Cell viability

Cell viability was determined using a WST-8 cytotoxicity assay (nacalai tesque, Kyoto, Japan), a modified MTT assay. Ten microliters of the Cell Count Reagent SF containing 5 mM WST-8 was added to each well of 96-well culture plates. After incubating for 1 h at 37 °C, the absorbance of each well was measured at 450 nm with a reference wavelength of 655 nm.

### Fluorescence imaging of lysosome

Cells were incubated with LysoTracker® Blue DND-22 (Life Technologies Japan Ltd., Tokyo, Japan) for 30 min at 37 °C. After washing cells with phosphate buffer saline, fluorescence images were captured using confocal laser microscopy (LSM-710, Carl Zeiss, Jena, Germany).

### Ag concentrations

Ultracentrifugation of the cellular lysate was used to obtain the supernatant (soluble fraction) and the pellet (insoluble fraction). The acid-digested samples were diluted with deionized water and the concentrations of Ag were measured by inductively coupled plasma-mass spectrometry (Model 7500c, Agilent Technologies, Tokyo, Japan) at m/z 107 as described previously [13].

### Quantitative real-time PCR

Total RNA was isolated using the RNeasy® Plus Kit (Qiagen Gmbh, Hilden, Germany). Aliquots of total RNA (1 μg) were reverse-transcribed into cDNA with the High Capacity cDNA Reverse Transcription Kits (Life Technologies Japan Ltd.) according to the manufacturer’s instructions. Quantitative real-time PCR analysis was performed using a StepOne Real-Time PCR System and the Universal SYBR® Select Master Mix (Life Technologies Japan Ltd.) as described previously [14]. The primer sequences were as follows: MT-I, 5’-CTTGGGATCTCCAACCTCAC-3’ (forward) and 5’-AGGTGCATTTGCACTCTTTG-3’ (reverse); MT-II, 5’-ATGGATCCCAACTGCTCCT-3’ (forward) and 5’-GCATTTGCACTCTTTGCATT-3’ (reverse); GAPDH, 5’-AATCCCATCACCATCTTCCA-3’ (forward) and 5’-TGGACTCCACGACGTACTCA-3’ (reverse). The expression levels of MT-I and MT-II mRNAs were normalized to the level of GAPDH expression.

### Statistics

Results are expressed as mean ± standard deviation. Statistical significance was determined with a one-way analysis of variance followed by the Dunnett’s multiple-comparison test. When two groups were compared, a Student’s t-test or Welch’s t-test was used. A value of *P* < 0.05 was considered statistically significant.

## Results and discussion

**Fig. 2:**
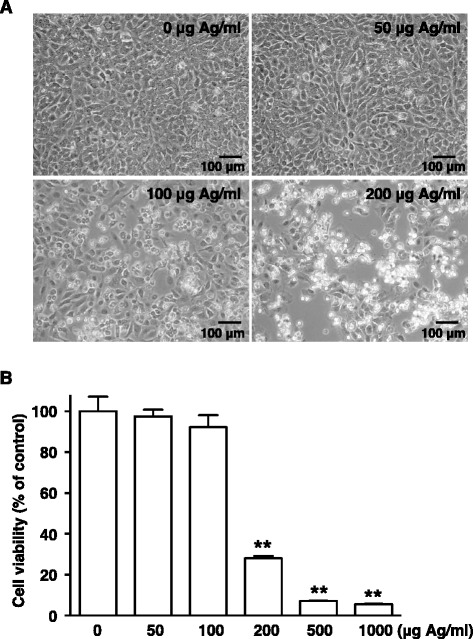
AgNP-induced cellular damage. **a** Phase-contrast micrographs. A549 cells were exposed to AgNPs (50–200 μg Ag/ml) for 24 h. Scale bar, 100 μm. **b** Cell viability. A549 cells were exposed to AgNPs (50–1000 μg Ag/ml) for 24 h. Cell viability was determined using a WST-8 assay. Each value (mean ± standard deviation, *n* = 3) represents the percent survival relative to untreated control cells (0 μg Ag/ml). **P < 0.01 compared to the control

**Fig. 3:**
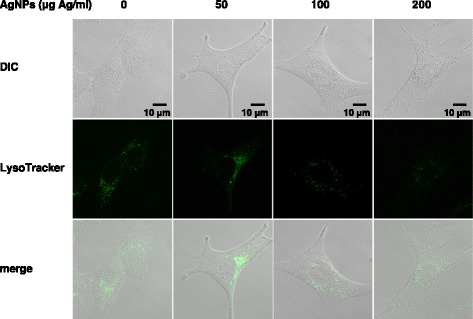
Colocalization of AgNPs with lysosomes and perturbation of lysosomes. A549 cells were exposed to AgNPs (50–200 μg Ag/ml) for 24 h and incubated with LysoTracker for 30 min. The lysosomes of cells were visualized by confocal laser microscopy with LysoTracker staining (green signal). Differential interference contrast (DIC) and fluorescence images were merged (merge). Scale bar, 10 μm

Exposure to AgNPs at the doses of 100 or 200 μg Ag/ml for 24 h caused A549 cells to separate from each other and to change from an epithelioid to a rounded shape (Fig. 2a). This cellular damage was found to be severe at the higher dose (200 μg Ag/ml). A WST-8 assay also showed a decrease in cell viability at 200 μg Ag/ml and for higher doses (Fig. 2b). We wanted to determine whether this cellular damage is caused by the action of AgNPs on lysosomes by using LysoTracker, a dye that accumulates in acidic vesicles [15]. Confocal laser microscopic examination of LysoTracker-stained A549 cells showed that AgNPs colocalized with lysosomes and their agglomeration increased in a dose-dependent manner (50–200 μg Ag/ml) (Fig. 3). On the other hand, the fluorescence signals of LysoTracker decreased as AgNP dose increased. These findings demonstrated that AgNP agglomerates accumulated in the lysosomes of lung epithelial cells in agreement with data from non-pulmonary cells exposed to AgNPs, such as human hepatoma cells [4], mesenchymal stem cells [5], HeLa cells [6], leukemia cells [7], and rat embryonic cells [8].

**Fig. 4:**
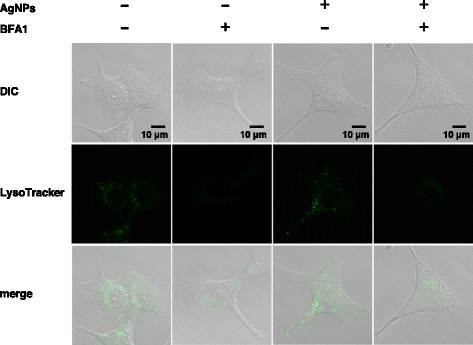
Effects of bafilomycin A1 treatment on AgNP-induced perturbation of lysosomes. A549 cells were incubated in culture medium with or without 200 nM bafilomycin A1 (BFA1) for 24 h before treating with or without AgNPs (50 μg Ag/ml) for an additional 24 h. Then, cells were incubated with LysoTracker for 30 min. The lysosomes of cells were visualized by confocal laser microscopy with LysoTracker staining (green signal). DIC and fluorescence images were merged (merge). Scale bar, 10 μm

**Fig. 5:**
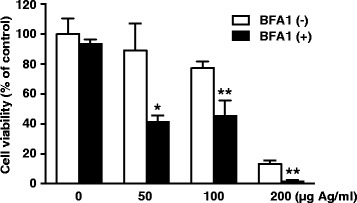
Effects of bafilomycin A1 treatment on AgNP-induced cell death. A549 cells were incubated in culture medium with or without 200 nM BFA1 for 24 h before treating with or without AgNPs (50–200 μg Ag/ml) for an additional 24 h. Cell viability was determined using a WST-8 assay. Each value (mean ± standard deviation, *n* = 4) represents the percent survival relative to untreated control cells (without AgNPs and BFA1). *P < 0.05, **P < 0.01 compared to cells without BFA1 at each concentration of AgNPs

**Fig. 6:**
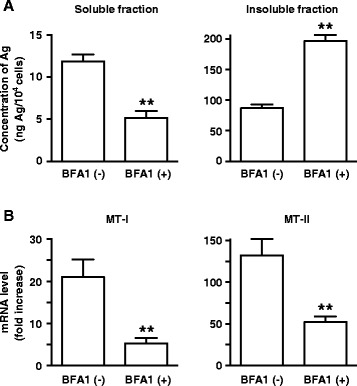
Effects of bafilomycin A1 treatment on Ag dissolution and metallothionein expression following exposure to AgNPs. A549 cells were incubated in culture medium with or without 200 nM BFA1 for 24 h before treating with AgNPs (50 μg Ag/ml) for an additional 24 h. **a** Ag concentrations in the soluble (left) and insoluble fractions (right). Values (ng/10^4^ cells) are mean ± standard deviation (*n* = 3). **b** mRNA levels of MT-I (left) and MT-II (right). Total RNA was isolated and subjected to real-time PCR analysis using MT-I or MT-II gene-specific primers. Each value (mean ± standard deviation, *n* = 3) was normalized to the GAPDH level, and the control value (cells without BFA1 and AgNPs) was set to 1. **P < 0.01 compared to cells without BFA1 and with AgNPs

It has been proposed that AgNPs are degraded to the Ag^+^ ion in the acidic lysosomal environment [16]. Our finding that cellular damage (Fig. 2) and AgNPs agglomeration were accompanied by decreased LysoTracker staining (Fig. 3) during exposure to varying doses of AgNPs raised the possibility that lysosomal dysfunction, represented by lysosomal alkalization, might play a role in AgNP cytotoxicity in A549 cells. Therefore, we examined the effects of lysosomal pH alkalization induced by bafilomycin A1 treatment in AgNP-exposed A549 cells. As expected, treatment of A549 cells with bafilomycin A1 (200 nM) alone reduced LysoTracker staining. AgNP (50 μg Ag/ml) and bafilomycin A1 treatment reduced the fluorescence signals of LysoTracker and increased AgNP agglomerates in A549 cells (Fig. 4). While treatment with bafilomycin A1 alone did not induce cell death, AgNP (50, 100, and 200 μg Ag/ml) treatment combined with bafilomycin A1 treatment significantly reduced cell viability (Fig. 5). When compared to A549 cells exposed to AgNPs (50 μg Ag/ml) alone, the concentration of Ag in the soluble fraction significantly decreased by 56 % and the concentration in the insoluble fraction increased by 125 % in cells treated with bafilomycin A1 and AgNPs (Fig. 6a), suggesting a decrease in the degradation of AgNPs to the Ag^+^ ion in lysosomes.

It has been reported that AgNPs incorporate into cells, and the Ag^+^ ion induces *de novo* synthesis of MT-I and MT-II [17], cytoprotective low-molecular weight proteins. Treatment with bafilomycin A1 significantly decreased the AgNP-induced (50 μg Ag/ml) elevation of MT-I and MT-II mRNA expression by 74 % and 61 %, respectively (Fig. 6b). Although the mechanism underlying fluctuation of the acidic lysosomal environment by AgNP exposure is not clear, our experiments using bafilomycin A1 further support the possible involvement of lysosomal dysfunction in AgNP cytotoxicity in A549 cells. In contrast to the proposed preferential role of released Ag^+^ ions [1, 16, 18, 19], our results suggest that AgNP agglomerates/aggregates and other nanoparticle-specific properties might also contribute to A549 cellular damage.

Lysosomes are considered to be critical intracellular organelles responsible for the cytotoxicity of nanomaterials [20, 21]. Similar to AgNPs, gold nanoparticles have been reported to increase lysosomal pH and induce lysosome impairment in normal rat kidney cells [22]. Therefore, lumenal alkalization by these metallic nanoparticles might lead to lysosomal dysfunction with cytotoxic consequences [20]. Furthermore, a neutral red assay showed lysosomal destabilization in AgNP-exposed hepatopancreas cells from adult oysters (*Crassostrea virginica*) [23]. Inhibition of cathepsin, a lysosomal protease, suppressed the release of interleukin-1β from human blood monocytes treated with AgNPs [24]. These findings suggest that AgNP exposure could disrupt the lysosomal-membrane, i.e., lysosomal-membrane permeabilization (LMP) [25]. Partial LMP induces apoptosis via mitochondrial outer membrane permeabilization, and massive LMP induces necrosis via cytosolic acidification [20]. Further investigation of lysosomal function will provide clues to understanding the mechanisms of pulmonary damage caused by exposure to AgNPs and other heavy metallic nanoparticles.

In summary, the present study presents data suggesting that lysosomal dysfunction caused by AgNP exposure may reduce pH-dependent Ag dissolution and MT expression, resulting in the cellular damage of pulmonary epithelial cells. Additional experiments, including animal models, are needed to reveal the precise mechanism of the perturbation of lysosomal function by AgNP exposure.

## Abbreviations

AgNPs: silver nanoparticles
BFA1: bafilomycin A1
DIC: differential interference contrast
DMSO: dimethyl sulfoxide
H^+^-ATPase: proton-pumping ATPase
LMP: lysosomal-membrane permeabilization
MT: metallothionein
TEM: transmission electron microscopy
